# Essential Oil and Major Non-Volatile Secondary Metabolites from the Leaves of Amazonian *Piper subscutatum*

**DOI:** 10.3390/plants10061168

**Published:** 2021-06-09

**Authors:** Jorge Ramírez, María Daniela Andrade, Giovanni Vidari, Gianluca Gilardoni

**Affiliations:** 1 Departamento de Química, Universidad Técnica Particular de Loja, Calle M. Champagnat s/n, Loja 1101608, Ecuador; jyramirez@utpl.edu.ec (J.R.); madandrade3@utpl.edu.ec (M.D.A.); 2 Dipartimento di Chimica, Università degli Studi di Pavia, Via Taramelli 10, 27100 Pavia, Italy; cistre@unipv.it; 3 Medical Analysis Department, Faculty of Science, Tishk International University, Erbil 44001, Iraq

**Keywords:** *Piper subscutatum*, *Artanthe scutata*, Piperaceae, essential oil, enantioselective analysis, 7,7′-epoxylignans, Ecuador

## Abstract

The essential oil and the major non-volatile secondary metabolites from the leaves of *Piper subscutatum* (Miq.) C. DC. (Family Piperaceae), collected in the Ecuadorian Amazon, were analyzed for the first time in the present study. The essential oil was submitted to chemical and enantioselective analyses by GC-MS and GC-FID. (*E*)-β-caryophyllene (25.3–25.2%), β-chamigrene (10.3–7.8%), (*E*)-nerolidol (8.1–7.7%), β-selinene (7.2–7.7%), δ-cadinene (2.7–3.9%), bicyclogermacrene (3.7–2.4%), and β-pinene (2.6–3.4%) were the major components. The enantioselective analysis, carried out on a β-cyclodextrin-based column, showed four scalemic mixtures in which (1*R*,5*R*)-(+)-α-pinene, (1*S*,5*S*)-(−)-β-pinene, (*S*)-(−)-limonene, and (1*R*,2*S*,6*S*,7*S*,8*S*)-(−)-α-copaene were the major enantiomers, with enantiomeric excesses of 28.8%, 77.8%, 18.4%, and 6.0%, respectively. The study was complemented with the chemical analysis of the organic fraction dissolved in the hydrolate, whose major components were 6-methyl-5-hepten-2-one (63.7–64.4%) and linalool (6.5–6.0%). Concerning the non-volatile fraction, five lignans were the major components. (–)-Beilshminol B, (–)-grandisin, (–)-3′,4′-methylenedioxy-3,4,5-trimethoxy-7,7′-epoxylignan, (–)-3′,4′-methylenedioxy-3,4,5,5′-tetramethoxy-7,7′-epoxylignan, and (–)-3,4,3′,4′-dimethylenedioxy-5,5′-dimethoxy-7,7′-epoxylignan were identified by means of NMR spectroscopy, mass spectrometry and X-ray crystallography. The absolute configuration 7*S*,8*S*,7′*S*,8′*S* was tentatively assigned to all of them.

## 1. Introduction

*Piper subscutatum* (Miq.) C. DC. is a rather common spontaneous shrub, belonging to the family Piperaceae, that is also known with the synonym *Artanthe scutata* Miq. The plant is distributed between Ecuador and Peru, where it has been described in the Amazonian regions of both countries. It ranges from 100 to approximately 2000 m above sea level [1]. *P. subscutatum* phytochemicals have not been investigated to date. 

Ecuador is located in the north-western coast of South America, laying between Colombia and Peru, with an area of approximately 283,500 Km^2^. From west to east, its territory can be divided into four geo-climatic areas, the islands (Galapagos), the coast, the highlands, and the forest. The equator line crosses the territory at about 24 km north of the capital Quito, passes through Isabela Island (Galapagos), and gives the name to the country. All these features are responsible for the presence of an outstanding biodiversity, with more than 5000 of the world’s plants as endemics, and a marine ecosystem existing within its borders. For these data, Ecuador is considered one of the 17 megadiverse countries of the Earth [2]. An update of phytochemical studies performed on Ecuadorian plants, until 2016, has been published recently [3]. Approximately 400 indexed papers are reported in this review, that concern more than 15,000 native botanical species. Interestingly, about 50% of the publications are related to only 8 botanical families over a total of about 250; as a result, most Ecuadorian plants are still an attractive source for phytochemical investigations.

In this context, more than 20 years ago, the authors of this work started a systematic study of characteristic plants of the Ecuadorian flora. As a result of their efforts, new essential oils have been described [4,5,6,7,8,9,10,11,12,13], the chemical structures of novel secondary metabolites have been established [14,15,16,17], and several biological activities have been determined [18,19,20,21]. In continuation of this phytochemical program, the present investigation of *Piper subscutatum* (Miq.) C. DC. deals with the composition of the essential oil (EO) and major non-volatile metabolites of the plant. It is worth noting that a complete de novo phytochemical study should, in principle, include both the volatile and non-volatile fractions, which is rarely done. In particular, the EOs are defined by the European Pharmacopoeia as “odorous products, usually of complex composition, obtained from a botanically defined plant raw material by steam-distillation, dry distillation, or a suitable mechanical process without heating. Essential oils are usually separated from the aqueous phase by a physical process that does not significantly affect their composition” [22].

In general, *Piper* species display a wide range of biological activities and produce a great variety of novel bioactive secondary metabolites, most of which are formed via the shikimic acid pathway [23,24,25,26]. A couple of *Piper* species, characteristic of the Ecuadorian flora, have been investigated by us in the past [6,21]. Now, we have extended our studies to *P. subscutatum*, which is a plant characteristic of the subtropical Ecuadorian flora and is quite abundant in the growth stations.

The aim of the study was to further contribute to the phytochemistry of the *Piper* species growing in Ecuador and, more generally, to the metabolic description of the Neotropical flora. Moreover, the search for new natural products or pharmacologically interesting compounds was considered equally important.

## 2. Results

### 2.1. Chemical Analysis of the EO and Hydrolate

The EO was steam distilled from fresh leaves of *P. subscutatum*, with a yield of 0.21% (*w/w*). Both qualitative and quantitative analyses were carried out on two orthogonal stationary phases: a non-polar DB-5ms column (polydimethylsiloxane with 5% phenyl groups) and a polar HP-INNOWax column (polyethylene glycol). In the qualitative analysis, a total of 66 oil components were identified by comparison of their mass spectra and linear retention indices with the literature. On the other hand, 60 compounds were quantified on at least one column, with a detection threshold of 0.1%. Quantified components corresponded to 96.9% and 95.1% of the EO total mass, on the non-polar and polar column, respectively. The sesquiterpene fraction was predominant in the EO, corresponding to 86.3% and 81.1% on the two columns, respectively. The major components, with an average amount ≥ 3% over the two columns, were (*E*)-β-caryophyllene (25.3–25.2%), β-chamigrene (10.3–7.8%), (*E*)-nerolidol (8.1–7.7%), β-selinene (7.2–7.7%), δ-cadinene (2.7–3.9%), bicyclogermacrene (3.7–2.4%), and β-pinene (2.6–3.4%). After solid phase extraction (SPE), the same semi-quantitative analysis was carried out on the hydrolate, that is, the aqueous phase spontaneously separating from the EO after the distillation. In this case, analytical results are better expressed as concentrations rather than percentages. Sulcatone (6-methyl-5-hepten-2-one) was the major organic solute in the hydrolate, with a concentration of about 64 mg/100 mL. All the analytical data are exposed in Table 1.

### 2.2. Enantioselective Analysis of the EO

The enantioselective analysis of the EO was carried out on a diethyl-*tert*-butyldimethylsilyl-β-cyclodextrin based capillary column. Four enantiomeric pairs were detected, comprising three monoterpenes and one sesquiterpene. The compounds were identified by their mass spectra and calculated linear retention indices (LRIs). The main enantiomers were (1*R*,5*R*)-(+)-α-pinene, (1*S*,5*S*)-(−)-β-pinene, (*S*)-(−)-limonene and (1*R*,2*S*,6*S*,7*S*,8*S*)-(−)-α-copaene, with enantiomeric excesses of 28.8%, 77.8%, 18.4% and 6.0%, respectively. The results are reported in Table 2. 

### 2.3. Lignans from P. Subscutatum Ethyl Acetate Extract

**Figure 1:**
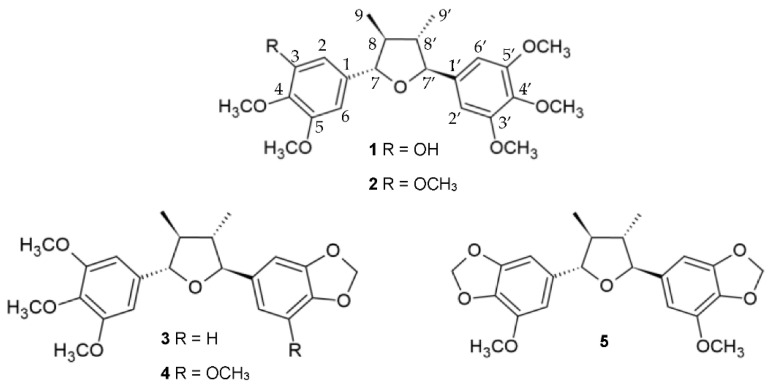
Lignans isolated from the ethyl acetate extract of *P. subscutatum*.

**Figure 2:**
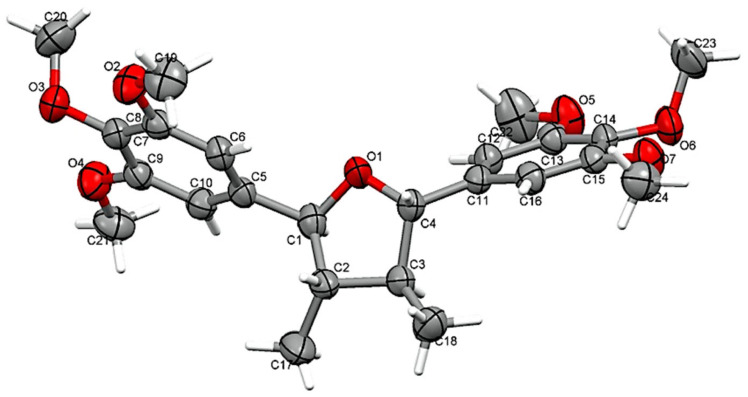
Computer generated Oak Ridge Thermal Ellipsoid Plot (ORTEP) structure (hydrogen atoms excluded) of (–)-grandisin (**2**) isolated from *P. subscutatum*.

**Figure 3:**
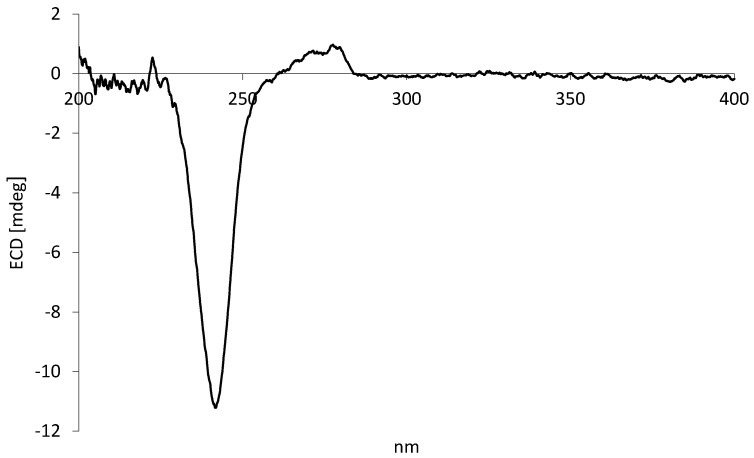
ECD spectrum of (–)-grandisin (**2**) isolated from *P. subscutatum*.

Five tetrahydrofuran lignans (Figure 1) were isolated by preparative column chromatographic separation of a chlorophyll-free EtOAc leaf extract of *P. subscutatum*. The molecular structures were determined by nuclear magnetic resonance (NMR) spectroscopy, mass spectrometry (MS) and, in the case of compound 2, by X-ray crystallography (Figure 2). Comparison of the spectral data with the literature clearly indicated that these lignans were identical to beilshminol B (1) [39], grandisin (2) [40], and three related compounds, 3′,4′-methylenedioxy-3,4,5-trimethoxy-7,7′-epoxylignan (3) [41], 3′,4′-methylenedioxy-3,4,5,5′-tetramethoxy-7,7′-epoxylignan (4) [42], and 3,4,3′,4′-dimethylenedioxy-5,5′-dimethoxy-7,7′-epoxylignan (5) [42]. The relative configuration *rel*-(7R,8R,7′R,8′R) was inferred for isolated compounds 1 and 3–5 on the basis of NOESY studies, and values of coupling constants and chemical shifts of protons 7, 7′, 8, 8′, 9, and 9′ in the ^1^H NMR spectra [39,40,41,42,43]. In fact, they were very similar to each other and to those of grandisin (2), whose relative configuration has firmly been established [40,43,44,45]. Moreover, the negative optical rotations and the negative ECD (electronic circular dichroism) peak (Figure 3) observed at about 245 nm, strongly suggested that compounds 1–5 had the same configuration at the four stereocenters. Despite these compounds having not yet surrendered to total synthesis, some closely structurally related 7,7′-epoxylignans, such as (–)-talaumidin, (+)-fragasin A_2_, (+)-galbelgin, (–)-galbelgin, (+)-galbacin, and (–)-galbacin, have been obtained in enantiomerically pure form by enantioselective synthesis [46]. In this context, it is important to note that the configuration 7*S*,8*S*,7′*S*,8′*S* was assigned to all the laevorotatory 2,3-*anti*-3,4-*anti*-4,5-*anti* configured 2,5-diaryl-3,4-disubstituted epoxylignans that are structurally related to compounds 1–5; in contrast, the dextrorotatory synthetic forms showed the opposite stereochemistry. Moreover, despite being much rarer than the laevorotatory enantiomer, (+)-grandisin is also a natural product and the configuration 7*R*,8*R*,7′*R*,8′*R* was assigned to this lignan [47]. Thus, based on this evidence, the absolute configuration 7*S*,8*S*,7′*S*,8′*S* was assigned to all the laevorotatory lignans 1–5. 

## 3. Discussion

The pattern of the EO components and non-volatile lignans appear to characterize *P. subscutatum* from a chemical point of view, significantly differentiating this plant from other *Piper* species [23,24,25,26,48]. In recent papers [6,26,49], the compositions of the EOs from the genus *Piper* have been discussed and oils were divided into six groups depending on the predominant chemical classes. Thus, according to this classification, the *P. subscutatum* EO belongs to the group dominated by sesquiterpenes, which contributed to more than 80% of the entire sample. Interestingly, the relative abundance (about 25%) of the major component (*E*)-β-caryophyllene was the same as that found for the EO of *Piper coruscans* [6]. (*E*)-β-Caryophyllene was also a dominant component of the EOs of *P. majusculum*, *P. madeiranum*, *P. duckei*, and *P. nigrum* [26]. The presence of sulcatone (6-methyl-5-hepten-2-one) among the volatile compounds of *P. subscutatum* is quite important, from a chemotaxonomic and biological point of view. In fact, this citrus-like fruity odorant has rarely been detected in the EOs of *Piper* species [50]. Due to its hydrophilicity, and despite the amount in the EO did not exceed 3%, it constituted more than 80% of the organic content in the hydrolate (up to 64.4 mg/100 mL). Furthermore, sulcatone, which is naturally emitted by the human body, is one of several mosquito attractants that are specific for hematophagous species such as *Aedes aegypti* [51]. This insect is responsible for the diffusion of many dangerous tropical viruses, causing dengue, yellow fever, chikungunya, and zika. Considering that all these diseases scourge the growing regions of *P. subscutatum*, where the plant can be collected in rather large amounts, the hydrolate obtained from this plant has the potential to be used as a natural pest trap by poor rural communities.

Concerning the non-volatile compounds **1–5** isolated from *P. subscutatum*, tetrahydrofuran lignans are typical secondary metabolites of *Piper* species, whose biosynthesis in plants proceeds via the shikimic acid pathway. Lignans **1** and **3–5** are rare lignans, that have only been isolated once in nature thus far. (–)-Beilshminol B (**1**) was isolated from the Chinese plant *Beilschmiedia tsangii* [39], whereas compound **3** was isolated from a Brazilian specimen of *Virola surinamensis* [41]. Finally, lignans **4** and **5** were both discovered in the Brazilian species *Piper solmsianum* [42]. (–)-Grandisin (**2**) is by far the most abundant lignan occurring in *P. subscutatum* leaves, accounting for about 14.5 % of the crude EtOAc extract. It is a rather common lignan, that was at first isolated in 1974 from *Litsea grandis* [44] and, subsequently, from several Piperaceae, Lauraceae, Myristicaceae and Schisandraceae species among others [43]. (–)-Grandisin has displayed several biological activities, such as trypanocidal activity against the trypomastigote form of *Trypanosoma cruzi*, larvicidal activity against the mosquito *Aedes aegypti*, and antinociceptive, anti-inflammatory, and antitumor activities [52]. Especially interesting are the remarkable trypanocidal activity of compounds **4** and **5** [42,53,54] against *Trypanosoma cruzi*, which is the aetiological agent of Chagas disease. In fact, according to the World Health Organization, about 6–7 million people in the world are estimated to be currently infected by this protozoan parasite, most of them living in South America, including Ecuador [55]. The early administration of synthetic compounds such as benznidazole and nifurtimox can eradicate the protozoan with very high efficiency. However, the lack of the drugs, as well as their late application in the countryside, make Chagas a major cause of death in rural regions of South America. Therefore, the availability of an effective and unexpensive anti-Chagas natural product must be considered a valuable alternative to synthetic drugs. In this context, a purified extract of *P. subscutatum*, enriched in lignans **2**, **4**, and **5**, should be tested in the field as an anti-Chagas agent.

## 4. Materials and Methods

### 4.1. General Information

The chemical and enantioselective analyses of the *P. subscutatum* EO were carried out with an Agilent Technologies GC-MS system, consisting of a 6890N gas chromatograph with an autoinjector model 7683. The instrument was coupled to an Agilent Technologies mass spectrometry detector (MSD) model 5973 INERT (Santa Clara, CA, USA), and a common flame ionization detector (FID). The MSD operated in SCAN mode (scan range 40–350 *m/z*), with an electron ionization (EI) source at 70 eV. The qualitative and quantitative analyses were run with both apolar and polar capillary columns. The apolar column was based on 5% phenyl methylpolysiloxane (DB-5ms from Agilent Technologies, 30 m long, 0.25 mm internal diameter, and 0.25 μm film thickness), whereas the polar column was provided with a polyethylene glycol stationary phase (HP-INNOWax, from Agilent Technologies, 30 m × 0.25 mm × 0.25 μm). The enantioselective analysis was performed with a capillary column, based on 30% diethyl-*tert*-butyldimethylsilyl-β-cyclodextrin on PS-086 as the chiral selector. The column was 25 m × 250 μm internal diameter × 0.25 μm phase thickness, and was purchased from Mega, MI, Italy. The carrier gas for all the analyses was GC purity grade helium (Indura, Guayaquil, Ecuador), set at the constant flow rate of 1 mL/min. Preparative chromatographic separations were performed on open columns at atmospheric pressure (CC) or by means of a medium pressure liquid chromatograph (MPLC) Reveleris^®^ Prep System (Büchi Labortechnik, Flawil, AG, CH), equipped with both a UV-vis and a light scattering detector. Columns packed with silica gel (Merck Kieselgel 60, 40–63 μm) or C_18_ reversed phase (Merck LiChroprep RP-18, 25–40 μm), both purchased from Sigma-Aldrich (St. Louis, MO, USA), were used. All TLC analyses were conducted over silica gel 60 (0.25 mm; GF_254_, Merck) or RP-18 (F_254s_, Merck) plates (Sigma-Aldrich). TLC spots were visualized under UV light (254 and 366 nm), followed by exposure to a 0.5% solution of vanillin in H_2_SO_4_/ethanol 4:1, and subsequently heated at 100 °C. For applications of solid phase extraction (SPE), the cartridges were standard products, packed with 1 g of C_18_ reversed phase and purchased from Sigma-Aldrich. For chlorophyll removal, SPE was carried out over a manually packed column. All NMR experiments were carried out with a Varian (400 MHz, Varian Inc., Palo Alto, CA, USA) spectrometer. The number of protons attached to each carbon was determined by DEPT experiments. Deuterated solvents were purchased from Sigma-Aldrich. Electron spray ionization mass spectrometry (ESI-MS) experiments were conducted by means of a Bruker Amazon Speed spectrometer (Bruker, Billerica, MA, USA). Optical rotations were measured on an Automatic Polarimeter (Jinan Hanon Instruments Co. Ltd., Jinan, China) MRC P810. ECD spectra were observed on a Jasco spectropolarimeter, model J-1100 (Jasco Co. Ltd., Japan). Melting points were determined with a Fisher-Johns apparatus (Thermo Fisher Scientific, Waltham, MA, USA). Single crystal X-ray diffraction experiments were carried out on an Enraf-Nonius four-circle diffractometer, model CAD-4 (Enraf-Nonius B.V., Rotterdam, NL), applying a graphite monochromated Mo-Kα radiation. The ORTEP structure of grandisin (**2**) (Figure 2) showed 50% probability displacement ellipsoids. Technical grade solvents (Brengtan, Guayaquil, Ecuador), freshly distilled, were used for preparative chromatographic separations, whereas HPLC grade solvents (Sigma-Aldrich) were used for all other applications. The mixture of *n*-alkanes C_9_–C_25_ and the internal standard (*n*-nonane) for GC analysis were of analytical grade (purity > 99%) and purchased from Sigma-Aldrich. The calibration standard was isopropyl caproate, synthesized in the authors’ laboratory and purified to 98.8% purity (GC-FID).

### 4.2. Plant Material

Leaves of *P. subscutatum* were collected at Numbani (Zamora-Chinchipe Province) in April 2018, under the supervision of one of the authors (J.R.), who also identified the botanical species. The collection was carried out under permit MAE-DNB-CN-2016-0048, issued by the Ministry of Environment of Ecuador (MAE). A botanical specimen was deposited at the herbarium of the Universidad Técnica Particular de Loja, with the voucher number PPN-pi-011. Equal amounts of ten different shrubs, which were growing homogeneously in a range of about 500 m around a central point (coordinates 4°09′24.9″ S and 78°56′38.9″ W), were collected. Hence, a total of ten equal plant samples were obtained. As all these specimens were representative of the same small ecologically and geologically homogeneous area, leaves were mixed, producing an average sample, which afforded both the EO and the EtOAc extract. The EO was distilled from fresh leaves the same day of collection, whereas solvent extraction was carried out on dried plant material. The drying process was conducted at 35 °C for three days, followed by storage of the vegetable material in the darkness, at room temperature, until use.

### 4.3. Distillation of the EO and GC Sample Preparation

Fresh leaves (2.6 kg) were steam distilled in a stainless-steel Clevenger-type apparatus for 4 h. The EO, that spontaneously separated from the aqueous layer, was removed with a pipette, dried over anhydrous sodium sulphate and stored in the darkness at −14 °C until use. Two volumes of hydrolate (10 mL each) were also collected and immediately processed to afford two analytical samples.

For each GC injection, 10 mg of the EO were weighted and diluted with 1 mL of cyclohexane, previously spiked with *n*-nonane (0.7 mg/mL) as the internal standard. Regarding the hydrolate analysis, each portion of the aqueous layer was eluted on a previously conditioned SPE cartridge. After complete removal of water from the solid phase, analytes were recovered by elution with 2 mL of acetone, previously spiked with the internal standard. The two acetone solutions were then directly injected separately into the GC apparatus.

### 4.4. GC-MS Qualitative Analyses

The EO and the hydrolate were analyzed by injecting 1 μL of each sample into the GC apparatus that operated in split mode (40:1). The injector temperature was set at 220 °C. Each sample was analyzed on both the polar and non-polar columns. With the DB-5ms column, oven temperature was set as follows: 50 °C (1 min isothermal), raised to 250 °C with a gradient rate of 3 °C/min, and then isothermal at 250 °C (10 min). With the polar column, the oven temperature program was the same, except that the final temperature was set at 230 °C. A homologous series of *n*-alkanes, from *n*-nonane to *n*-pentacosane, was injected in each column to calculate the linear retention index (LRI) of each analyte [56]. Each volatile metabolite was identified by comparing the corresponding LRI value and EI-MS spectrum with the literature (see Table 1).

### 4.5. GC-FID Quantitative Analyses

All the samples were injected in duplicate in a GC-FID chromatograph, under the same conditions and configurations as the GC-MS analyses. The percentage of each analyte in the EO and the hydrolate was calculated as the average value over the two analyses. The relative response factor (RRF) of each analyte was calculated on the basis of the corresponding combustion enthalpy [57,58]. Two calibration curves (one for each column), both with r^2^ = 0.999, were obtained as described in a previous study [6] and applied to all the samples.

### 4.6. Enantioselective Analysis of the EO

The enantioselective analysis of the EO was carried out by injecting the sample into the gas-chromatograph in the GC-MS configuration. The injector temperature and split ratio were the same as for the EO qualitative analysis, whereas oven temperature was set as follows: 50 °C (5 min isothermal), then raised to 220 °C with a gradient rate of 2 °C/min, and then isothermal at 220 °C (5 min). The enantiomeric pairs of chiral terpenes were identified on the basis of the EI-MS spectra and the order of elution, as described in previous studies [4,5,6].

### 4.7. Preparation of the Ethyl Acetate Extract

An amount (250 g) of *P. subscutatum* dried leaves were minced and then macerated in ethyl acetate for one hour at room temperature. The process was repeated three times and the resulting extracts were combined, filtered, and distilled under vacuum at 35 °C, to afford 4.19 g of an oily residue. Chlorophyll was then removed by SPE, passing the residue through a column packed with C_18_ reversed phase (50 g). Elution with 80% aqueous methanol gave, after evaporation, 1.76 g of a chlorophyll-free residue.

### 4.8. Separation of the Lignans

The chlorophyll-free extract (1.0 g) was separated by MPLC on a column packed with commercial C_18_ reversed phase (100 g). Elution with a gradient from 65% aqueous MeOH to 100% MeOH, over 60 min, afforded, after TLC control over C_18_ reversed phase plates, 13 main fractions, named from JR-01 to JR-13.

Fraction JR-04 (71.9 mg) was separated on a column packed with silica gel (7 g), at atmospheric pressure. Isocratic elution with a mixture of *n*-hexane-EtOAc, 90:10, afforded compound **1** (7.3 mg).

Pure (–)-grandisin (**2**, 610 mg) spontaneously crystallized in the collection tube of fraction JR-05. The crystals were separated by filtration and washed with *n*-hexane before X-ray analysis.

Fraction JR-07 (82 mg) was separated on a column packed with silica gel (8 g), at atmospheric pressure. Isocratic elution with *n*-hexane-EtOAc, 85:15, gave compounds **3** (5.7 mg) and **4** (18.6 mg).

Finally, fraction JR-08 (27.3 mg) was separated on a column packed with silica gel (3 g), at atmospheric pressure. Isocratic elution with *n*-hexane-EtOAc, 95:5, afforded compound **5** (3.3 mg). 

(–)-*Beilshminol B* (***1***).

C_23_H_30_O_7_: pale yellowish oil; ${\left[\alpha \right]}_{D}^{20}=-24.8$ (*c* 0.015, CH_2_Cl_2_). ESI-MS (*m/z*): 418.41 [M]^+^, 441.41 [M+Na]^+^, 859.36 [2M+Na]^+^. ^1^H NMR (400 MHz, CDCl_3_): δ_H_ 1.06 and 1.08 (2d, *J* = 6.0 Hz, 2 × 3H, H_3_-9,-9’), 1.75-1.85 (m, 2 × 1H, H-8,-8’), 3.84 (s, 3H, C*H*_3_O-4’), 3.88 (s, 4 × 3H, C*H*_3_O-3’,-4,-5,-5’), 4.62-4.66 (2d, *J* = 9.0 Hz, 2 × 1H, H-7,-7’), 6.55 (d, *J* = 2.0 Hz, 1H, H-6), 6.62 (s, 2 × 1H, H-2’,-6’), 6.64 (d, *J* = 2.0 Hz, 1H, H-2). ^13^C NMR (100 MHz, CDCl_3_): δ_C_ 14.1 (*C*H_3_-9’), 14.2 (*C*H_3_-9), 51.1 (*C*H-8’), 51.2 (*C*H-8), 56.1 (*C*H_3_O-5), 56.3 (*C*H_3_O-3’,-5’), 61.0 (*C*H_3_O-4’), 61.1 (*C*H_3_O-4), 88.4 (*C*H-7), 88.7 (*C*H-7’), 101.9 (*C*H-6), 103.0 (*C*H-2’), 103.1 (*C*H-6’), 105.9 (*C*H-2), 134.9 (C-4), 137.5 (C-4’), 138.1 (C-1’), 138.9 (C-1), 149.2 (C-3), 152.6 (C-5), 153.4 (C-3’,-5’). The spectral data nicely correspond to the literature [39]. 

(–)-*Grandisin* (***2***).

C_24_H_32_O_7_: colourless crystals; m.p.: 120–122 °C; ${\left[\alpha \right]}_{D}^{20}=-43.6$ (*c* 0.140, CH_2_Cl_2_); ECD_242 nm_ (c 0.03, MeOH) = −11.21 mdeg. ESI-MS (*m/z*): 433.53 [M+H]^+^, 455.51 [M+Na]^+^, 888.03 [2M+Na]^+^. ^1^H NMR (400 MHz, CDCl_3_): δ_H_ 1.08 (d, *J*=6.2 Hz, 2 × 3H, H_3_-9,-9’), 1.70-1.85 (m, 2 × 1H, H-8,-8’), 3.85 (s, 2 × 3H, C*H*_3_O-4,-4’), 3.90 (s, 4 × 3H, C*H*_3_O-3,-3′,-5,-5′), 4.65 (d, *J*= 9.5 Hz, 2 × 1H, H-7,-7’), 6.62 (s, 2 × 2H, H-2,-2’,-6,-6’). ^13^C NMR (100 MHz, CDCl_3_): δ_C_ 14.1 (*C*H_3_-9,-9′), 51.1 (*C*H-8,-8′), 56.2 (*C*H_3_O-3,-5,-3,-5′), 60.9 (*C*H_3_O-4,-4′), 88.6 (*C*H-7,-7′), 103.1 (*C*H-2,-6,-2′,-6′), 137.4 (C-4,-4′), 138.0 (C-1,-1′), 153.2, 153.3 (C-3,-5, -3′,-5′). The spectral data nicely correspond to the literature [40,43,44]. 

(–)-(*7S,8S,7′S,8′S*)-*3′,4′-Methylenedioxy-3,4,5-trimethoxy-7,7′-epoxylignan* (***3***).

C_22_H_26_O_6_: pale yellow oil; ${\left[\alpha \right]}_{D}^{20}=-27.9$ (*c* 0.020, CH_2_Cl_2_). ESI-MS (*m/z*): 387.14 [M+H]^+^, 409.14 [M+Na]^+^, 795.11 [2M+Na]^+^. ^1^H NMR (400 MHz, CDCl_3_): δ_H_ 1.05 and 1.10 (2d, *J* = 6.0 Hz, 2 × 3H, H_3_-9,-9’), 1.70-1.85 (m, 2 × 1H, H-8,-8’), 3.85 (s, 3H, C*H*_3_O-4), 3.90 (s, 2 × 3H, C*H*_3_O-3,-5), 4.62 (d, *J* = 10.0 Hz, 2 × 1H, H-7,-7’), 5.94 (s, 2H, OC*H*_2_O), 6.61 (s, 2 × H, H-2,-6), 6.78 (d, *J* = 8.0 Hz, 1H, H-5′), 6.86 (dd, *J* = 8.0 and 1.5 Hz, 1H, H-6′), 6.93 (d, *J* = 1.5 Hz, 1H, H-2′). The spectral data nicely correspond to the literature [41]. 

(–)-(*7S,8S,7′S,8′S*)-*3′,4′ -Methylenedioxy-3,4,5,5′-tetramethoxy-7,7′-epoxylignan* (***4***).

C_23_H_28_O_7_: pale yellow oil; ${\left[\alpha \right]}_{D}^{20}=-44.3$ (*c* 0.270, CH_2_Cl_2_). ESI-MS (*m/z*): 417.33 [M+H]^+^, 439.23 [M+Na]^+^, 855.25 [2M+Na]^+^. ^1^H NMR (400 MHz, CDCl_3_): δ_H_ 1.04 (2 overlapped d, *J* = 6.0 Hz, 2 × 3H, H_3_-9,-9’), 1.73-1.78 (m, 2 × H, H-8,-8’), 3.84 (s, 3H, C*H*_3_O-4), 3.88 (s, 2 × 3H, C*H*_3_O-3,-5), 3.92 (s, 3H, C*H*_3_O-5′), 4.60 (d, *J* = 8.0 Hz, 2 × 1H, H-7,-7′), 5.95 (s, 2H, OC*H*_2_O), 6.60 and 6.62 (2 × s, 2 × 1H each, H-2,-6,-2′,-6′). The NMR data correspond to the literature [42].

(–)-(*7S,8S,7′S,8′S*)-*3,4,3′,4′-Dimethylenedioxy-5,5′-dimethoxy-7,7′-epoxylignan* (***5***).

C_22_H_24_O_7_: pale yellow oil; ${\left[\alpha \right]}_{D}^{20}=-19.8$ (*c* 0.019 in CH_2_Cl_2_). ESI-MS (*m/z*): 401.25 [M+H]^+^, 423.25 [M+Na]^+^, 823.50 [2M+Na]^+^. ^1^H NMR (400 MHz, CDCl_3_): δ_H_ 1.04 (d, *J* = 6.2 Hz, 2 × 3H, H_3_-9,-9’), 1.73-1.78 (m, 2 × 1H, H-8,-8′), 3.91 (s, 2 × 3H C*H*_3_O-5,5′), 4.58 (d, *J* = 9.0 Hz, 2 × 1H, H-7,-7′), 5.96 (s, 2 × 2H, 2 × OC*H*_2_O), 6.59 (s, 4 × 1H, H-2,-6,-2′,-6′). The NMR data correspond to the literature [42].

## 5. Conclusions

The phytochemical profile and the leaf EO components of the Ecuadorian species *Piper subscutatum* (Miq.) C. DC. have been determined in the present study for the first time. In addition to a new sesquiterpene-based essential oil, produced in a fairly substantial yield, *P. subscutatum* afforded some other potentially useful products. Thus, the hydrolate, a cheap by-product of the EO process, contained a considerable amount of sulcatone that is a well-known mosquito attractant. On the other hand, three highly active trypanocidal lignans were isolated from the non-volatile fraction. Therefore, it appears that *P. subscutatum*, which can be collected in rather large amounts in the wild, may have a remarkable practical importance, as some active products, isolable from the plant, can be used to fight *A. aegypti* and other dangerous insects infesting tropical countries. Clearly, these applications should be confirmed by future field studies.

## Figures and Tables

**Table 1:** Qualitative and quantitative chemical analyses of the EO and hydrolate from *P. subscutatum* fresh leaves.

Compounds	DB-5ms		HP-INNOWax		EO (%) ^2^		Hydrolate (mg/100 mL)	
	LRI ^1^	LRI [27]	LRI ^1^	LRI	DB-5ms	HP-INNOWax	DB-5ms	HP-INNOWax
α-pinene	925	932	1014	1025 [28]	1.6	2.1	--	--
camphene	939	946	1071	1075 [28]	trace	trace	--	--
β-pinene	968	974	1103	1118 [28]	2.6	3.4	--	--
6-methyl-5-hepten-2-one (sulcatone)	983	981	1339	1323 [29]	2.1	2.9	63.7	64.4
myrcene	986	988	1163	1166 [30]	0.2	0.6	--	--
α-phellandrene	1001	1002	1159	1162 [29]	0.3	0.3	--	--
δ-3-carene	1003	1008	1142	1135 [28]	0.2	trace	--	--
α-terpinene	1011	1014	1174	1175 [31]	trace	trace	--	--
*ƿ*-cymene	1019	1020	1268	1281 [28]	trace	trace	--	--
limonene	1023	1024	1194	1196 [29]	0.6	0.7	--	--
1,8-cineol	1026	1026	1201	1212 [29]	0.1	0.2	--	--
1,4-cineol	--	--	--	--	--	--	0.4	0.5
γ-terpinene	1052	1054	1242	1254 [28]	trace	trace	--	--
*p*-mentha-2,4(8)-diene(isoterpinolene)	1078	1085	1279	--	1.8	2.1	--	--
*trans*-linalool oxide (furanoid)	--	--	--	--	--	--	1.1	1.1
linalool	1100	1095	1555	1554 [31]	1.1	1.4	6.5	6.0
β-ylangene	--	--	1563	1576 [32]	--	trace	--	--
1,3,8-*p*-menthatriene	1126	1108	1421	1411 [32]	trace	0.3	--	--
borneol	--	--	--	--	--	--	0.3	0.7
terpinen-4-ol	--	--	--	--	--	--	0.5	0.5
α -terpineol	--	--	--	--	--	--	0.8	--
linalool formate	--	--	--	--	--	--	0.2	0.4
δ-elemene	1326	1335	1465	1468 [32]	0.2	0.2	--	--
α-cubebene	1337	1348	1451	1460 [32]	0.3	0.3	--	--
β-copaene	--	--	1579	1579 [31]	--	4.7	--	--
α-copaene	1362	1374	1481	1493 [33]	0.9	1,0	--	--
β-cubebene	1376	1387	1530	1541 [32]	trace	0.6	--	--
β-elemene	1379	1389	1574	1580 [34]	1.6	0.2	--	--
cyperene	1385	1398	1511	1528 [32]	0.1	0.1	--	--
sibirene	1392	1400	1528	--	1.7	0.3	--	--
α-gurjunene	1397	1409	1518	1529 [32]	0.2	1.7	--	--
(*E*)-β-caryophyllene	1404	1417	1587	1598 [32]	25.3	25.2	--	--
β-gurjunene	1414	1431	1601	1596 [32]	0.2	trace	--	--
α-guaiene	1423	1437	1632	1652 [32]	1.9	0.2	--	--
α-cedrene	1428	1410	1594	1600 [35]	0.2	0.6	--	--
6,9-guaiadiene	1433	1442	1621	1617 [36]	0.3	0.3	--	--
α-humulene	1438	1452	1657	1666 [32]	1.8	0.4	--	--
myltayl-4(12)-ene	1442	1445	1622	--	0.4	0.2	--	--
ishwarene	1447	1466	1677	--	1.2	0.3	--	--
4,5-di-*epi*-aristolochene	1454	1471	1669	--	0.3	1.4	--	--
α-*neo*-clovene	1459	1452	1667	--	1.7	1.7	--	--
*cis*-cadina- 1(6),4-diene	1462	1461	1680	--	0.6	0.6	--	--
*cis*-muurola-4(14),5-diene	1465	1465	1651	1643 [32]	1.4	trace	--	--
selina-3,7(11)-diene	--	--	1762	1783 [32]	--	trace	--	--
β-selinene	1472	1489	1706	1716 [32]	7.2	7.7	--	--
viridiflorene	1476	1496	1685	1696 [32]	1.6	1.7	--	--
γ-himachalene	--	--	1716	1708 [32]	--	0.6	--	--
β-chamigrene	1480	1476	1712	1723 [32]	10.3	7.8	--	--
*trans*-cadina-1(6),4-diene	1483	1475	1697	--	0.2	1.4	--	--
α-bulnesene	1488	1509	1627	1629 [32]	1.4	1.7	--	--
bicyclogermacrene	1490	1500	1722	1734 [32]	3.7	2.4	--	--
*trans*-muurola-4(14),5-diene	1498	1493	1700	--	0.3	0.6	--	--
γ-cadinene	1502	1513	1783	1763 [32]	0.7	0.1	--	--
δ-cadinene	1506	1521	1750	1755 [32]	2.7	3.9	--	--
*trans*-calamenene	1508	1522	1825	1823 [32]	1.1	0.7	--	--
*trans*-cadina-1,4-diene	1518	1533	--	--	0.2	--	--	--
7-*epi*-α-selinene	1524	1520	1766	1764 [32]	0.2	0.4	--	--
*cis*-muurol-5-en-4-β-ol	--	--	1182	--	--	0.2	--	--
α-calacorene	1525	1544	1907	1921 [32]	0.7	0.5	--	--
β-germacrene	1539	1559	1815	1823 [32]	1.8	1.4	--	--
(*E*)-nerolidol	1558	1561	2048	2036 [32]	8.1	7.7	--	--
caryophyllene oxide	1563	1582	1967	1986 [32]	0.2	0.5	--	--
cubebol	--	--	1934	1941 [32]	--	0.2	--	--
*trans*-dauca-4(11),7-diene	1569	1556	1726	--	0.5	0.2	--	--
cubenol	--	--	2066	2067 [32]	--	0.4	--	--
guaiol	1586	1600	2075	2090 [37]	0.2	0.2	--	--
1-*epi*-cubenol	1612	1627	2087	2088 [32]	0.4	trace	--	--
*epi*-α-cadinol	1630	1638	2166	2169 [32]	0.2	0.2	--	--
α-muurolol (torreyol)	1633	1644	2171	2183 [32]	0.2	trace	--	--
10-*epi*-γ-eudesmol	1637	1622	2093	2105 [32]	1.5	0.2	--	--
pogostol	1641	1651	2182	2196 [38]	2.5	0.2	1.1	0.1
α-eudesmol	--	--	--	--	--	--	0.8	0.3
5-*neo*-cedranol	1679	1684	2191	--	0.1	0.2	--	--
*trans*-pinocarveol	--	--	--	--	--	--	--	0.2
neral	--	--	--	--	--	--	--	0.5
nerol	--	--	--	--	--	--	--	0.2
Monoterpene hydrocarbons					7.3	9.5	--	--
Oxygenated monoterpenes					3.3	4.5	73.5	73.6
Sesquiterpene hydrocarbons					72.9	71.1	--	--
Oxygenated sesquiterpenes					13.4	10,0	1.9	1.3
Total					96.9	95.1	75.4	74.9

^1^ Calculated linear retention index; ^2^ Trace for % <0.1.

**Table 2:** Enantioselective analysis of chiral components of *P. subscutatum* EO, on a chiral diethyl-*tert*-butyldimethylsilyl-β-cyclodextrin-based column.

Enantiomers	LRI ^1^	Enantiomeric Ratio	*ee ^2^* (%)
(1*R*,5*R*)-(+)-α-pinene	927	64.4	28.8
(1*S*,5*S*)-(−)-α-pinene	928	35.6	
(1*R*,5*R*)-(+)-β-pinene	953	11.1	77.8
(1*S*,5*S*)-(−)-β-pinene	960	88.9	
(*S*)-(−)-limonene	1052	59.2	18.4
(*R*)-(+)-limonene	1067	40.8	
(1*R*,2*S*,6*S*,7*S*,8*S*)-(−)-α-copaene	1663	53.0	6.0
(1*S*,2*R*,6*R*,7*R*,8*R*)-(+)-α-copaene	1666	47.0	

^1^ Calculated linear retention index; ^2^ enantiomeric excess.

## Data Availability

Raw data are available from the authors (J.R.).
